# Intelligence

**Published:** 1828-11

**Authors:** 


### INTELLIGENCE.

### MONTHLY REPORT OF PREVALENT DISEASES.

During the whole of the last month, pneumonic diseases have been very common, especially in children. In a few cases we have found adults labouring under a similar affection, which has generally been combined with rheumatism in one or other of the extremities. Moderate bleedings, aperients, and expectorants, have been successfully adopted. In two instances of the complaint, which has been almost epidemic amongst children, recovery appeared to be retarded by too free bleeding at the commencement. Blisters to the chest have not appeared in any instance decidedly beneficial, and, in more than one, children have suffered much from the general irritation they produced.

Three cases of severe periodical headache have occurred to us. They were effectually relieved by purging at first, and the subsequent employment of the sulphate of quinine, in one-grain doses three times a day. During the paroxysm the forehead was freely bathed with eau de Cologne, with much advantage.

*Apothecaries' Hall.—Regulations for the Examination of Apothecaries.*—The Court of Examiners chosen and appointed by the Master, Wardens, and Assistants of the Society of Apothecaries of the City of London, in pursuance of a certain Act of Parliament, "for better Regulating the Practice of Apothecaries throughout England and Wales," passed in the fifty-fifth year of the reign of his Majesty King George the Third, apprise all persons whom it may concern:

That every candidate for a certificate to practise as an apothecary will be required to possess a competent knowledge of the Latin language, and, in compliance with the 14th and 15th sections of the said Act, to produce testimonials of having served an apprenticeship* of not less than five years to an apothecary; of having attained the full age of twenty-one years, and being of good moral conduct.

* Articles of apprenticeship, where such are in existence, will be required; but in case such articles shall have been lost, it is expected that the candidate shall bring forward very strong testimony to prove that he has served such an apprenticeship, as the Act of Parliament directs.

He will also be required to produce certificates of having attended not less than—

Two courses of lectures on Chemistry;

Two courses of lectures on Materia Medica and Botany;

Two courses of lectures on Anatomy and Physiology;

Two courses of Anatomical Demonstrations.

Two courses of lectures on the Theory and Practice of Medicine:* these last to be attended subsequently to one course of lectures on Materia Medica, Chemistry, and Anatomy;

And a certificate† of attendance for six months,‡ at least, on the physicians' practice of some public hospital or infirmary, (containing not less than sixty beds,) or for nine months at a dispensary: such attendance to commence subsequently to the termination of the first course of lectures on the Principles and Practice of Medicine.

The regulations relating to the order of succession in which the lectures on the Practice of Medicine, and the physicians' practice of an hospital or dispensary, are to be attended, are designed to apply to those students only who commenced their attendance on lectures on or after the 1st of February, 1828; and all such persons are particularly requested to take notice, that, unless they shall have strictly complied with such order of succession, they will not be admitted to an examination.

In addition to the course of study above required as indispensably necessary, candidates are earnestly recommended to attend clinical lectures, and also lectures on Midwifery and the Diseases of Women and Children; on the latter of which subjects, as an important part of medical practice, they will be examined.

The Court have determined, that the examination of the candidate shall be as follows:

1. In translating grammatically parts of the Pharmacopœia Londinensis and physicians' prescriptions; and, after the 1st of January, 1831, candidates will be required to translate portions of the following medical Latin authors, viz. "Celsus de Medicina," or "Gregory Conspectus Medicinæ Theoreticæ."

2. In Chemistry.

3. In the Materia Medica.

4. In Botany.

5. In Anatomy and Physiology.

6. In the Practice of Medicine.

*Notice.*—Every person intending to qualify himself, under the regulations of this Act, to practise as an apothecary, must give notice in writing, ad-

* No testimonial of attendance on lectures on the Principles and Practice of Medicine, delivered in London, or within seven miles thereof, will render a candidate eligible for examination, unless such lectures were given, and the testimonial is signed by, a Fellow, Candidate, or Licentiate, of the Royal College of Physicians.

† Physicians' pupils, who intend to present themselves for examination, must appear personally at the beadle's office, in this Hall, and bring with them the tickets, authorising their attendance on such practice, as the commencement thereof will be dated from the time of such personal appearance.

‡ All candidates applying for examination after the 1st of October, 1829, will be required to produce evidence of having attended the physicians' practice at an hospital or infirmary for nine months, or at a dispensary for twelve months.

dressed to the clerk of the Society, on or before the Monday previously to the day of examination; and must also at the same time deposit all the required testimonials at the office of the beadle at Apothecaries' Hall, where attendance is given every day (except Sunday) from nine until two o'clock.

Persons intending to present themselves for examination are requested to take notice, that they may obtain, at the beadle's office at this Hall, a printed paper containing certificates, with blanks (as to names and dates) of all the lectures they are required to have attended, and also of the physicians' practice. These blanks the Court request may be filled up and signed by the respective lecturers, and by the physicians whose practice the student has attended.

Students are enjoined to observe, that, after the 1st of November, 1828, these certificates, so filled up, will be required from candidates for examination. After the same day, no other testimonials of attendance on lectures and medical practice will be admitted, except such as bear the seal of a university or college, and the signature of an officer belonging to such university or college whose duty it is to sign certificates of attendance on the lectures given therein; or such other certificates as have heretofore been received, if the same were obtained prior to the 1st of February, 1828.

The Court will meet in the Hall every Thursday, where candidates are requested to attend at half-past one o'clock.

By order of the Court,

JOHN WATSON, *Secretary.*

*London; September 25th*, 1828.

Information relative to the business of this Court may be obtained of Mr. Watson, at his residence, 43, Berners-street, between the hours of nine and ten o'clock every morning, (Sunday excepted.)

*** It is expressly ordered by the Court of Examiners, that no gratuity be received by any officer from any persons applying for information relative to the business of this court.

---

*Biography of* Dr. Gall.—Dr. F. J. Gall was born in 1758, at Tubingen, a small town in the duchy of Baden, which, in the year 1805, was erected into the kingdom of Wurtemberg. In his boyhood he was distinguished by his extraordinary talent of observation, and by the profundity of his knowledge of natural objects. His disposition for abstruse reflection made him but of little interest to youths of his own age, so that he was generally either associated with companions much his seniors, or was to be found in the fields collecting birds' nests, flowers, or shells.

His father, wishing to turn his mind towards medicine, sent him to Vienna, where he graduated, and where he became a medical practitioner. It was here, during his studies, that he was first impressed with the remarkable want of information on the anatomy and functions of the nervous system; and here he made the researches upon which he based his simple and beautiful view of the uses of the different parts of the brain. It was not till after nearly twenty years of the first conception of the new theory of the functions of the brain, that Dr. Gall made known his opinions to the world. His first lectures on the anatomy and physiology of the brain and nervous system were delivered at Vienna, in the year 1796.

In 1805, Dr. Gall made a scientific tour of the north of Germany, accompanied by his friend and pupil Dr. Spurzheim, who was born at Longuich, on the Mozelle, in 1776. Dr. S. was associated with Dr. Gall in his labours for several years, and contributed greatly to elucidate the views of Dr. Gall, by his superior knowledge of the anatomy of the brain. He has since collected their discoveries; and, from the rude mass of facts, digested the most simple and most rational doctrine of mental philosophy. As it may be easily imagined, the promulgation of opinions so widely different from the favorite theory of the schools, created a very powerful sensation among the sçavans. Dr. Gall was, however, well received. To use his own words—" I met every where with a reception the most flattering: sovereigns, ministers, judges, philosophers, and artists, seconded my design of augmenting my collection of facts on every occasion. These circumstances were too favorable to allow me to resist the invitation I received from most of the universities. Hence it resulted that numerous public and private discussions took place upon my doctrine; and hence my notions acquired a greater degree of maturity than the founders of new doctrines have generally experienced. After having, during thirty years, employed means most diversified of examining the deductions I had made from facts innumerable, I feared neither personal danger nor reproach, but precipitated my great work upon the world."

Political events, about this period, absorbed every other consideration; and the rapid strides that Napoleon was making towards the helm of Europe left but little tranquillity to the secluded philosopher. Nor did this extraordinary man allow the new philosophy of the mind to go unconsidered: it pleased him, and he spoke of it in high terms; but the Jesuits of France, imagining that the doctrines favored materialism, exerted all their power to extinguish it in its birth. Its range was, however, too extensive for their power.

Dr. Gall then visited England, and ultimately made Paris his place of residence, where he became an eminent physician. Of late years, he gave public courses of lectures on his science at his own house, which were numerously attended, and his rooms became the resort of the sçavans of all Europe.

Dr. Gall had a fit of apoplexy a few weeks before his death, which took place on the 22d of August last. The funeral was accompanied by a vast number of eminent literary characters. MM. Broussais, Fossati, Fontanelli, Laudrer, Bourdon, and Vimond, held funeral orations; and M. Paillet de Ceombieres spoke some verses near the tomb.

Most of us find some amusement in tracing on fancy's tablet the portrait of a person of whom we have heard much, and particularly after we have read many of the works of an author, but with whom we have had no personal acquaintance. It generally happens, however, that our portrait is not correct, when compared with the original: thus it was with myself. I found Dr. Gall (in 1826) to be a man of middle stature, of an outline well proportioned; he was thin and rather pallid, and possessed a capacious head and chest. The peculiar brilliancy of his penetrating eye left an indelible impression. His countenance was remarkable, his features strongly marked and rather large, yet devoid of coarseness. The general impression that a first glance was calculated to convey would be, that Dr. Gall was a man of originality and depth of mind, possessing much urbanity, with some self-esteem and inflexibility of design.

After presenting my letters of introduction to him, at seven o'clock in the morning, he shewed me into a room, the walls of which were covered with bird-cages, and the floor with dogs, cats, &c. Observing that I was surprised at the number of his companions, he observed, "All you Englishmen take me for a bird-catcher. I am sure you feel surprised that I am not somewhat differently made to any of you, and that I should employ my time talking to birds."—"Birds, sir, differ in their dispositions like men; and, if they were but of more consequence, the peculiarity of their characters would have been as well delineated."—"Do you think," said he, turning his eyes to two beautiful dogs at his feet, which were endeavouring to gain his attention: "do you think that these little pets possess pride and vanity like man?"—"Yes," I said, "I have remarked their vanity frequently."—"We will call both feelings into action," said he. He then caressed the whelp, and took it into his arms: "Mark his mother's offended pride," said he, "as she walked quietly across the chamber to her mat: "do you think she will come if I call her?"—"Oh, yes," I answered.—"No, not at all." He made the attempt, but she heeded not the hand she had so earnestly endeavoured to lick but an instant before. "She will not speak to me today," said the Doctor.

He then described to me the peculiarity of many of his birds, and I was astonished to find that he seemed familiar also with their dispositions, (if I may be allowed the word.) "Do you think a man's time would be wasted thus in England? You are a wealthy and powerful nation, and, as long as the equilibrium exists between the two, so shall you remain; but this never has existed, nor can, beyond a certain period. Such is your industry, stimulated by the love of gain, that your whole life is spun out before you are aware the wheel is turning; and so highly do you value commerce, that it stands in the place of self-knowledge and an acquaintance with nature and her immense laboratory."

I was delighted with his conversation: he seemed to me to take a wider view in the contemplation of man than any other person with whom I had ever conversed. During breakfast, he frequently fed the little suitors, who approached as near as their iron bars would admit. "You see they all know me," said he, "and will feed from my hand, except the blackbird, who must gain his morsel by stealth before he eats it: we will retire an instant, and in our absence he will take the bread." On our return, we found he had secreted it in a corner of his cage. I mention these, otherwise uninteresting, anecdotes, to shew how much Dr. Gall had studied the peculiarities of the smaller animals.

After breakfast, he shewed me his extensive collection, which I find is purchased by an Englishman; and thus ended my first visit to the greatest moral philosopher that Europe has produced,—to a man, than whom few were ever more ridiculed, and few ever pursued their bent more determinately, despite its effects,—to a man who alone effected more change in mental philosophy than perhaps any predecessor,—to a man who suffered more persecution, and yet possessed more philanthropy, than most philosophers.

In comparing the characters of two men who, from their associated labours, are generally spoken of at the same time, we might say of Dr. Gall that he possessed the greater genius, while Dr. Spurzheim is the most acute reasoner. To the former we are indebted for the discovery of a new doctrine, to the latter for its adaptation to useful purposes. Gall astonished us by the vastness of his scheme of mental philosophy, Spurzheim by the attractions with which he adorned it. Gall possessed the genius that commands respect, and Spurzheim the amiability of disposition that ever ensures it.

*Birmingham; 20th Sept.* 1828. E. A. T.

### METEOROLOGICAL JOURNAL,

*From September 20th, to October 20th,* 1828.

By Messrs. HARRIS and Co. Mathematical Instrument Makers, 50, High Holborn.

| September | Rain gauge. | Moon. | Thermom. | | | Barometer. | | De Luc's Hygrom. | | Winds. | | Atmospheric Variations. | | |
|---|---|---|---|---|---|---|---|---|---|---|---|---|---|---|
| | | | 9 A.M. | MAX. | MIN. | 9 A.M. | 10P.M. | 9A.M. | 10P.M. | 9 A.M. | 10P.M. | 9 *a.m.* | 2 *p.m.* | 10 *p.m.* |
| 20 | | | 53 | 67 | 50 | 30.06 | 30.07 | 50 | 50 | SE | SE | Fine | Fine | Fine |
| 21 | | | 58 | 67 | 51 | .02 | .04 | 49 | 48 | SE | SSE | — | — | — |
| 22 | | | 58 | 66 | 58 | 29.75 | 29.84 | 47 | 49 | S | SW | Foggy | — | — |
| 23 | | ○ | 60 | 68 | 56 | 30.00 | 30.01 | 50 | 51 | SW | SW | Foggy | Fine | — |
| 24 | | | 61 | 69 | 59 | .01 | 29.97 | 51 | 52 | W | WSW | Fine | — | — |
| 25 | | | 62 | 68 | 60 | 29.85 | .75 | 51 | 47 | W | SW | — | — | — |
| 26 | | | 65 | 73 | 56 | .67 | .72 | 51 | 49 | SW | WNW | — | — | — |
| 27 | .60 | | 64 | 72 | 50 | .76 | .71 | 54 | 53 | NW | NW | Rain | Rain | Cloudy |
| 28 | .45 | | 60 | 67 | 51 | .60 | .50 | 53 | 52 | W | NW | Show'ry | — | Rain |
| 29 | | | 62 | 65 | 54 | .45 | .43 | 51 | 56 | WNW | WNW | Cloudy | Cloudy | Cloudy |
| 30 | | ☾ | 56 | 62 | 52 | .56 | .57 | 54 | 54 | W | W | Fine | Fine | Cloudy |
| Oct. | | | | | | | | | | | | | | |
| 1 | | | 58 | 66 | 48 | .48 | .57 | 55 | 54 | W | W | Cloudy | Fine | Fine |
| 2 | | | 56 | 61 | 45 | .68 | .81 | 55 | 54 | W | NW | Fine | — | Fine |
| 3 | | | 46 | 62 | 54 | .83 | .72 | 53 | 52 | WNW | SW | Foggy | — | — |
| 4 | .08 | | 60 | 63 | 54 | .43 | .53 | 52 | 54 | SSW | W | Show'ry | Cloudy | Cloudy |
| 5 | | | 57 | 64 | 53 | .31 | .37 | 53 | 53 | SW | SW | Fine | — | Cloudy |
| 6 | | | 57 | 60 | 50 | .22 | .41 | 52 | 54 | W | WSW | Fine | Cloudy | Fine |
| 7 | .07 | | 54 | 61 | 52 | .52 | .41 | 54 | 54 | W | W | Fine | Fine | Show'ry |
| 8 | | ● | 56 | 60 | 50 | .41 | .57 | 51 | 51 | WNW | NW | Fine | Cloudy | Cloudy |
| 9 | | | 56 | 60 | 48 | .81 | .96 | 51 | 54 | NW | NW | Fine | Fine | Fine |
| 10 | | | 53 | 60 | 51 | 30.00 | .95 | 53 | 55 | W | W | — | Cloudy | Clondy |
| 11 | | | 53 | 60 | 52 | .14 | 30.25 | 56 | 55 | WNW | WNW | — | Fine | Fine |
| 12 | | | 56 | 62 | 50 | .29 | .32 | 54 | 54 | W | W | Fine | — | — |
| 13 | | | 51 | 60 | 50 | .27 | .21 | 55 | 52 | W | NNW | Foggy | Fine | Foggy |
| 14 | | | 52 | 57 | 52 | .23 | .24 | 54 | 52 | NW | NNW | Fine | — | Fine |
| 15 | | | 55 | 59 | 51 | .21 | .19 | 54 | 53 | NNW | NNW | Fine | — | Fine |
| 16 | | ☽ | 54 | 63 | 52 | .17 | .18 | 55 | 54 | NNW | NNW | Foggy | Cloudy | Foggy |
| 17 | | | 56 | 59 | 45 | .03 | .00 | 54 | 55 | NW | NNW | — | Fine | Cloudy |
| 18 | | | 46 | 55 | 45 | .19 | .17 | 54 | 50 | ENE | E | Fine | Fine | Fine |
| 19 | | | 47 | 54 | 39 | .04 | 29.99 | 50 | 52 | E | E | Fine | Fine | Fine |

The quantity of Rain fallen in the month of September, was 2 inches and 32.100ths.

